# Rail Corrugation Index Development by Sound-Field Excitation on the Carriage Floor of In-Service Train

**DOI:** 10.3390/s23177539

**Published:** 2023-08-30

**Authors:** Wei-Lun Hsu, Chia-Ming Chang

**Affiliations:** 1 Department of Systems Engineering and Naval Architecture, National Taiwan Ocean University, Keelung 202301, Taiwan; 2 Department of Civil Engineering, National Taiwan University, Taipei 10617, Taiwan; changcm@ntu.edu.tw

**Keywords:** rail corrugation, onboard sensing deployment, vibration measurement, short-time Fourier transform, time-frequency analysis

## Abstract

The steel rail and wheel in the railway system offer a high precision and smooth-running surface. Nevertheless, the point of contact between the rail and wheel presents a critical area that can give rise to rail corrugation. This phenomenon can potentially elevate sound and vibration levels in the vicinity considerably, necessitating advanced monitoring and assessment measures. Recently, many efforts have been directed towards utilizing in-service trains for evaluating rail corrugation, and the evaluation has primarily relied on axle-box acceleration (ABA). However, the ABA measurements require a higher threshold for vibration detection. This study introduces a novel approach to rail corrugation detection by carriage floor acceleration (CFA), aimed at lowering the detection threshold. The method capitalizes on the acceleration data sensed on the carriage floor, which is induced by the sound pressure (e.g., sound-field excitation) generated at the wheel–rail contact point. An exploration of the correlation between these datasets is undertaken by simultaneously measuring both ABA and CFA. Moreover, a pivotal aspect of this research is the development of the eigenfrequency rail corrugation index (E-RCI), a mechanism that culminates energy around specific eigenfrequencies by CFA. Through this index, a focused analysis of rail corrugation patterns is facilitated. The study further delves into the stability, repeatability, and sensitivity of the E-RCI via varied measurement scenarios. Ultimately, the CFA-based rail corrugation identification is verified, establishing its practical applicability and offering a distinct approach to detecting and characterizing rail corrugation phenomena. This study has introduced an innovative methodology for rail corrugation detection using CFA, with the principal objective of lowering the detection threshold. This approach offers an efficient measurement technique for identifying rail corrugation areas, thereby potentially reducing maintenance costs and enhancing efficiency within the railway industry.

## 1. Introduction

The steel rail and wheel system is widely used in railway systems because this combination yields smooth and stable transportation. Steel provides high precision and a smooth-running surface for both the rail and wheel. However, this system has a sharp and critical area where the steel rail and wheel come into contact, and this area experiences high stress. When the train passes, a range of phenomena occurs within the wheel–rail contact area, including wheel–rail stick–slip and creep. These phenomena can lead to the formation of wavelength-fixed irregularities, commonly known as corrugation [1].

Rail corrugation can significantly increase the sound and vibration levels in the surrounding environment, and this corrugation requires sophisticated monitoring for development. The causes of rail corrugation are varied and can generally be classified into six types, including pinned-pinned, rutting, unsprung mass track interaction (P2), heavy haul, light rail, and trackform-specific [2,3]. Generally, the corrugation patterns observed in different train systems or different types of trains tend to have varying rail corrugation types.

In metro systems, rail inspections are frequently conducted within specified maintenance windows to minimize disruptions to regular train operations. These inspection activities are typically scheduled during the limited late-night hours. However, these inspections heavily rely on visual assessment methods, which can be significantly influenced by lighting conditions and the physiological state of maintenance personnel, among other factors. Additionally, corrugation analysis trolleys are utilized to measure the detailed wavelength of rail corrugation [2,4]. To enhance inspection efficiency and capture the interaction among the wheel, train speed, and rail corrugation, onboard measurements have been implemented for detecting rail corrugation. These measurements include methods like carriage interior noise analysis [5,6], computer vision techniques [7], laser triangulation [8], laser displacement measurement [9], and the notably prevalent approach of utilizing acceleration measurements [6,10,11,12,13,14].

The widely adopted method for acceleration measurement involves the placement of accelerometers on the axle-box of the wheel. Subsequently, the acquired acceleration data are employed to categorize track defects [10,11,12,13]. The collected axle-box acceleration data was processed using empirical mode decomposition and ensemble empirical mode decomposition, which proved effective in accurately detecting rail surface defects. Moreover, measurements of axle-box acceleration, carriage interior vibration, and noise were conducted in [6]. It shows when a high-speed train runs on different rail corrugation curve sections, the interior vibration coincides with axle-box acceleration by rail corrugation and demonstrates the carriage interior noise capability in identifying the rail corrugation. This demonstrates the capability of carriage interior noise in identifying rail corrugation. In the same way, the axle-box acceleration of high-speed trains has been utilized to inspect the characteristics of rail corrugation [14]. To avoid being affected by randomness, the rail corrugation index and energy factor were proposed as evaluation metrics for rail corrugation. 

**Figure 1:**
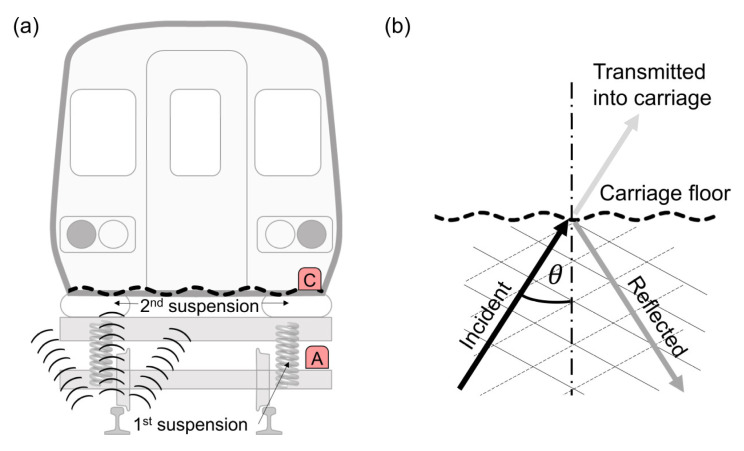
Sound pressure generated form wheel–rail contact point: (**a**) the relative position of the sound source and carriage floor; (**b**) incident sound wave on the carriage floor (transmitted into a carriage is not the subject of this study).

Axle-box acceleration (ABA) is widely used to evaluate rail corrugation because railway vehicles are equipped with two-level suspension systems (as shown in Figure 1a). The eigenfrequency of these suspension systems is usually at a significantly low frequency. As a result, relatively high-frequency vibration signals cannot pass through the suspension systems effectively. However, the ABA measurements require a higher threshold for vibration detection. 

This study introduces a novel approach to rail corrugation detection by carriage floor acceleration (CFA), aimed at lowering the detection threshold. When a train passes through a rail corrugation section, the wheel–rail contact point generates sound pressure (noise), which can be highly irritating. This sound pressure not only affects the surroundings but also manifests as vibrations transmitted to the carriage floor. This phenomenon, namely sound-field excitation, is a significant aspect, and therefore, this study first investigates sound-field excitation to inform and identify rail corrugations. In this study, the CFA is acquired to detect the sound pressure under the carriage. To accurately capture the eigenfrequency of rail corrugations, time-frequency analysis is employed by integrating the power spectral density (PSD) spectrogram with the singular value decomposition (SVD) method, resulting in the eigenfrequency-based rail corrugation index (E-RCI). The ABA and CFA will be measured simultaneously to investigate the correlation between the two data sets, and the E-RCI will be verified through in situ rail corrugation measurements.

To further validate the stability and repeatability of E-RCI in identifying rail corrugations, the case study includes the assessment of E-RCI using both the same and different train set CFA. Additionally, the sensitivity of E-RCI is evaluated by comparing the measurements before and after rail grinding, i.e., a maintenance procedure used to remove surface irregularities on the rail, including corrugations. The study aims to establish an easily executable and accurate E-RCI as a reliable tool for identifying, monitoring, and evaluating rail corrugations by in-service trains by conducting these comprehensive assessments and verifications. Ultimately, the CFA-based rail corrugation identification is verified, establishing its practical applicability and offering a distinct approach to detecting and characterizing rail corrugation phenomena. 

## 2. Sound-Field Excitation 

Railway noise and vibration predominantly originate from the point of contact between the wheel and rail. Moreover, the amalgamation of wheel and rail running surface roughness gives rise to vibrations, resulting in sound emission, commonly referred to as rolling noise [15]. 

The noise and vibration generated from the wheel–rail contact point disseminate throughout the vicinity. The vibrations ascend towards the wheel and axle-box (i.e., point A in Figure 1a) through direct transmission via the solid steel material. However, the eigenfrequencies of rail corrugations, particularly of the rutting corrugation type, are mostly above 250 Hz. Such high-frequency vibration energy does not pass through the first and second suspension systems due to their low natural frequencies, usually below 5 Hz [16].

On the contrary, the sound pressure generated from the wheel–rail contact point at a specific eigenfrequency will disseminate throughout all surrounding areas, including exciting the entire carriage floor, as shown in Figure 1a. The carriage floor is typically constructed of thin aluminum alloy plates, and the sound wave originating from the bottom of the carriage will excite the aluminum alloy floor plate, a phenomenon known as “sound-wave excitation”. This phenomenon has a resemblance to the transmission of sound through partitions, a well-known acoustic problem [17].

The problem of sound-wave excitation can be illustrated in Figure 1b. The incident sound wave generated from the wheel–rail contact point will excite the carriage floor plate at an angle of *θ*. The incident wave angle *θ* is always below 90°, which yields the wavenumber on the plate, denoted as $k_{p}$, to be smaller than that in the transmitted field, represented as $k_{a}$. Thus, the radiation ratio is defined [18] and written by

(1) $$\sigma_{rad}=\frac{1}{\sqrt{1-{\left(\frac{k_{p}}{k_{a}}\right)}^{2}}}=\frac{1}{\sqrt{1-sin^{2}\theta }},$$

Equation (1) shows that the radiation ratio $\sigma_{rad}$ is not corresponding to the frequency and is always above or equal to unity. Therefore, the measurement of sound pressure and vibration in the carriage can effectively capture the characteristics of sound pressure beneath the rigid carriage floor. Additionally, the wavelength induced on the plate wave must be equal to the wavefront of the sound. Therefore, the accelerometer mounted on the carriage floor (i.e., point C in Figure 1a) can effectively “hear” the sound pressure generated from the wheel–rail contact point beneath the carriage floor. The following studies will further verify the feasibility of utilizing CFA to evaluate rail corrugation. 

## 3. Data Supervisory and Acquisition System

**Figure 2:**
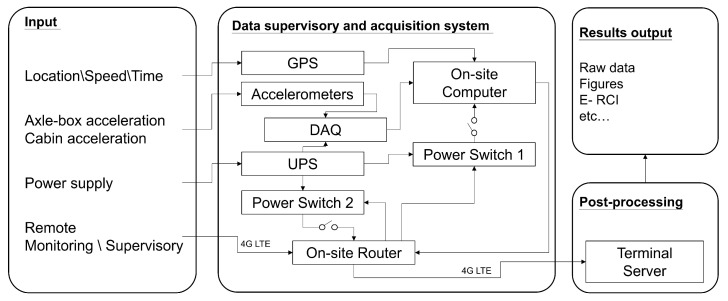
The layout of the in-service train data supervisory and acquisition system.

To monitor the long-term trend of rail corrugation, a data supervisory and acquisition system layout is shown in Figure 2. The ABA and CFA are simultaneously measured with the global positioning system (GPS) and localizing acceleration data, such as curve tangent-spiral points, stations, crossovers, and other relevant points.

**Figure 3:**
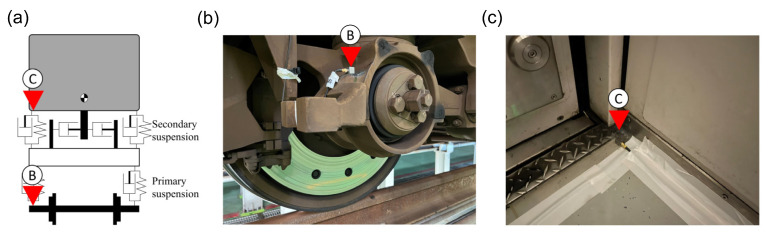
The accelerometers’ installed position on the in-service train: (**a**) the relative position of ABA and CFA measured on the train; (**b**) ABA measure accelerometer; (**c**) CFA measure accelerometer.

The accelerometers were installed on the in-service train, as illustrated in Figure 3a. The axle-box accelerometer was positioned on the gauge side of the train, marked as “B” in Figure 3b. Similarly, the carriage floor accelerometer was mounted at the door side of the carriage, just above the last axle of the train, indicated as “C” in Figure 3c. The specifications of the accelerometers and data acquisition system (DAQ) used in this study are detailed in Table 1. The sampling rate was set to 2000 Hz, with a resolution of 24 bits.

## 4. Measurement Data and Corrugation Identification Algorithm

### 4.1. Rail Corrugation in the Target Section 

**Figure 4:**
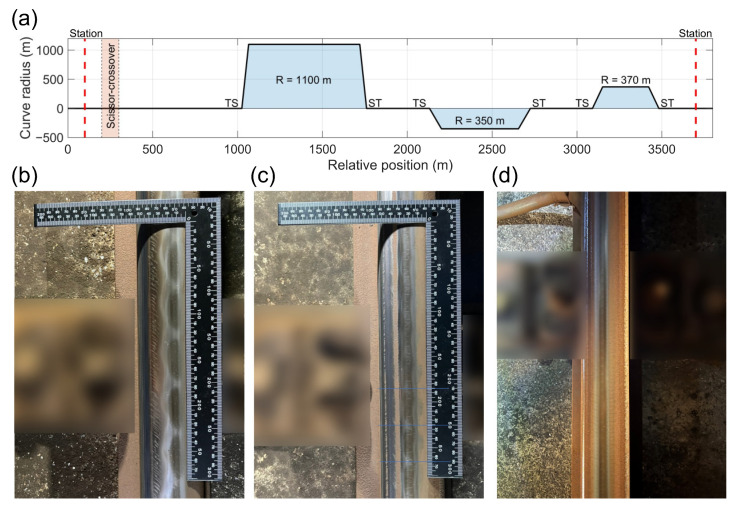
Recorded photo of rail corrugation by site visit: (**a**) curve and relative position of the target section; (**b**) rail condition at the first curve (R = 1100 m); (**c**) rail condition at the second curve (R = 350 m); (**d**) rail condition at the third curve (R = 300 m).

The vast majority of rail corrugations existed in the metro system′s curves in this study. The selected route section, referred to as the “target section”, as shown in Figure 4a, includes a scissor crossover and three curves with radii of 1100 m, 350 m, and 370 m, respectively. This section was selected strategically to encompass both larger and smaller curves, offering a comprehensive representation of the phenomenon under investigation in the study. Additionally, the train speed within the target section varies significantly as the train passes through these three curves. 

By the site visit, a thorough inspection of the rail surface condition at the first curve revealed the presence of rail corrugation, characterized by a wavelength of approximately 60 mm (as shown in Figure 4b). Notably, the corrugations were predominantly located on the high rail, specifically on the outer side of the curve. The rail corrugation was observed starting from the beginning of the curve at the tangent-spiral point (TS), and continued up to the end of the curve at the spiral-tangent point (ST). Upon proceeding to the next curve with an R = 350 m radius, it was observed that the rail corrugation was absent at the beginning of the curve. However, intriguingly, the corrugation emerged in the second half of the curve, exhibiting a distinct wavelength of approximately 40 mm (as depicted in Figure 4c). Finally, upon reaching the third curve with an R = 370 m radius, the rail surface presented itself in a smooth and impeccable state, devoid of corrugations (as shown in Figure 4d). This comprehensive site visit provided valuable insights into the distribution and nature of rail corrugations along the curves and served as a basis for further development and verification of the rail corrugation index. In the following section, the rail corrugation index developed in this study will be compared with the rail condition investigated during the site visit. 

### 4.2. The Measurement Data and Corresponding Spectrogram

**Figure 5:**
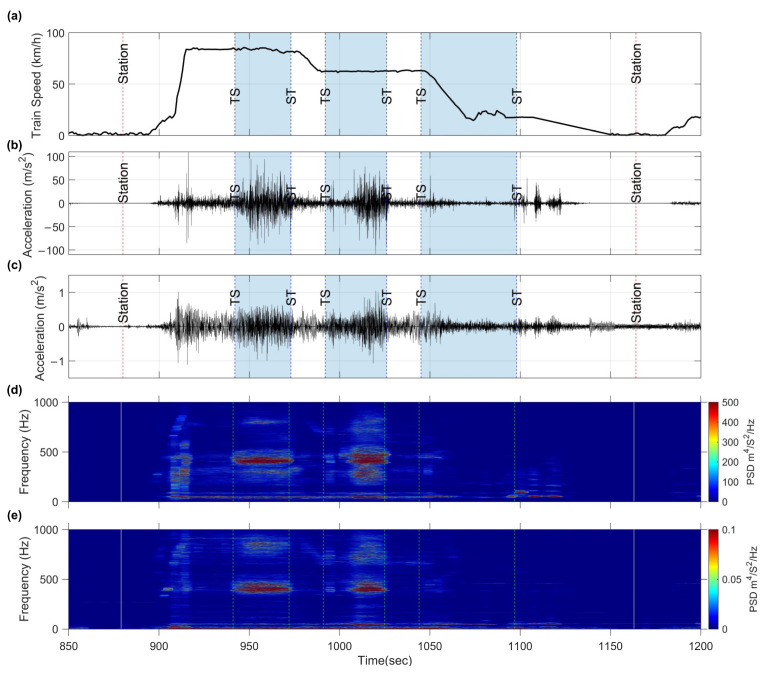
Measured time history and PSD spectrogram when the train passes through the target section: (**a**) train speed recorded by GPS; (**b**) the ABA time history data; (**c**) the CFA time history data; (**d**) the spectrogram of ABA; (**e**) the spectrogram of CFA. (The time history data were filtered by a 500 Hz lowpass filter yielding a distinct presentation.)

The train′s speed within the target section, as shown in Figure 5a, remains at 83 km/h while traversing the first curve. However, it subsequently decreases to 62 km/h upon entering the second curve. Finally, the speed further reduces in the third curve from 62 km/h to 17 km/h. This diverse speed profile is valuable in comprehending the presence of rail corrugation at different curve sections.

The time history of ABA and CFA data is presented in Figure 5b,c, respectively. A notable difference in the maximum acceleration values can be observed, with ABA reaching approximately 80 m/s² and CFA reaching 1.1 m/s^2^. Notably, when the train passes through a crossover, both accelerations experience a significant increase. However, when the train passed through the third curve, no significant increase in acceleration was observed in both measurement accelerometers.

To gain further insight into the significance of the time domain data, it is transformed into the frequency domain through PSD analysis. The PSD is represented by the spectrogram shown in Figure 5d,e. The spectrogram is obtained using the short-time Fourier transform (STFT) defined as follows:

(2) $$\mathrm{STFT}\left(t,\omega \right)=\int_{-\infty }^{\infty }x\left(t\right)w\left(t-t^{*}\right)e^{-i\omega t}dt$$

where $x\left(t\right)$ is the measured one-dimensional discrete acceleration signal, $w\left(t-t^{*}\right)$ is the moving window function shifted by $t^{*}$, and ω denotes the frequency in radians. As a result, the PSD spectrogram is constructed with m seconds and n frequencies. 

The features of the ABA and CFA spectrograms exhibit a high consistency. Notably, both the axle-box and carriage acceleration spectrograms share prominent peak frequencies at 430 Hz and 398 Hz, respectively. This alignment in the spectrogram characteristics confirms the hypothesis outlined in the “sound-field excitation” section. Despite the wave energy being transmitted through different routes, the measured accelerations emanate from the same source. In the subsequent section, the spectrogram will be quantified using the algorithm to identify the corrugation index.

### 4.3. Rail Corrugation Identification Algorithm by Carriage Floor Acceleration Data

The PSD spectrogram of axle-box and carriage floor accelerations reveals eigenfrequencies that correspond to the periodic wavelength of the rail surface. However, relying solely on the spectrogram is not an efficient approach to assessing the extent and severity of the rail corrugation. As a result, the rail corrugation index is developed in this study to provide a more effective means of quantifying the phenomenon. As discussed earlier, the sound and vibration induced by rail corrugation exhibit distinctive frequency features that contribute to their distinct appearances.

**Figure 6:**
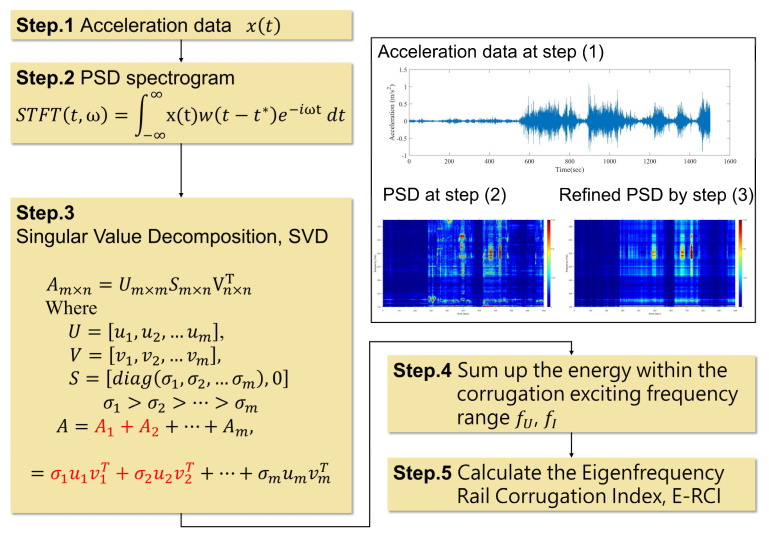
Flowchart of the corrugation identification algorithm.

Therefore, the rail corrugation index developed in this study focuses on capturing the energy around the eigenfrequencies, denoted as the eigenfrequency rail corrugation index (E-RCI). To ensure that the index is not affected by unrelated signals, the features of the PSD spectrogram are extracted using the singular value decomposition (SVD) method [19]. The flowchart of the proposed E-RCI development algorithm is presented in Figure 6. 

First, the PSD spectrogram was created, resulting in a matrix A with dimensions of m seconds and n frequencies. The SVD can be applied to matrix A, which can be defined as follows:

(3) $$A_{m\times n}=U_{m\times m}S_{m\times n}V_{n\times n}^{T},$$

where $U=\left[u_{1},u_{2},\ldots u_{m}\right]$, and $V=\left[v_{1},v_{2},\ldots v_{n}\right]$ are orthogonal matrixes. Moreover, U and V are eigenvectors of $AA^{T}$, and $A^{T}A$. The matrix S is diagonal, and the singular values are the entries of it, arranged in descending order. For the relationship, the matrix A can be decomposed as

(4) $$A=A_{1}+A_{2}+\cdots +A_{m},\;=\sigma_{1}u_{1}v_{1}^{T}+\sigma_{2}u_{2}v_{2}^{T}+\cdots +\sigma_{m}u_{m}v_{m}^{T},$$

By selecting a subset of the singular values and their associated singular vectors, a truncated matrix $A^{*}$ can be obtained. This refined matrix $A^{*}$ encapsulates the essence of the PSD spectrogram and can be useful for further analysis. 

Once the salient features of the PSD spectrogram have been developed, the upper bound $f_{U}$ and lower bound $f_{L}$ frequencies will be defined, tailored to the specific rail corrugation types and the desired wavelength identification. By accumulating the energy within the frequency range bounded by $f_{U}$, and $f_{L}$, and moving the time window, a time-depended E-RCI can be astutely derived. Moreover, to ensure impartiality and enhance comparability across different train sets, the E-RCI values will be thoughtfully normalized by the highest corrugation index value obtained from the specific train set, effectively mitigating any peculiarities related to the individual train conditions and offering a more robust baseline for comprehensive comparisons.

### 4.4. Comparison of Carriage and Axle-Box Corrugation Index

In this study, the in-service train acceleration is measured to discern the rail corrugation. Generally, the frequency induced by the uneven wave exhibits a linear correlation, which can be expressed as follows:

(5) f=V/λ,

where V is train speed in m/s, and λ is the rail corrugation wavelength in meters. 

**Figure 7:**
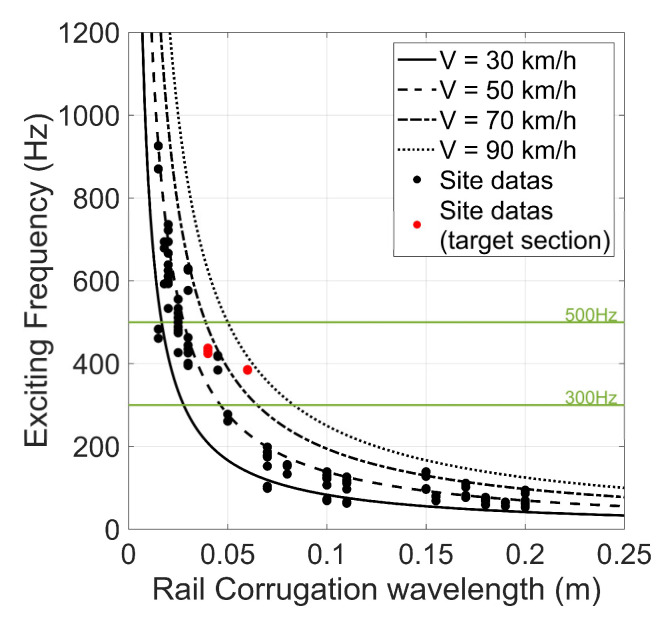
The exciting frequency in the relationship between train speed and rail corrugation wavelength.

As shown in Figure 7, when the train passes at specific speeds, the corresponding feature frequency is generated based on the variation in rail corrugation wavelength. The black dots in Figure 7 represent the relationship between rail corrugation wavelength investigated through site visits and its corresponding feature frequency, calculated using Equation (5) while considering the corresponding train′s speed. 

The red dots in Figure 7 represent the feature frequency generated by specific train speeds in the target section. As mentioned, the rail corrugation wavelength survey within the target section is mostly between 60 mm to 40 mm. When the train speed is between 83 km/h to 62 km/h, determined using GPS data, the exciting frequency should fall within the range of 384 Hz to 430 Hz, which closely aligns with the feature frequencies observed in Figure 5 spectrogram. Therefore, to identify the rail corrugation in the target section. In this study, the upper bound $f_{U}$ is set as 500 Hz and the lower bound $f_{L}$ as 300 Hz, encompassing the eigenfrequency range associated with the rail corrugation.

**Figure 8:**
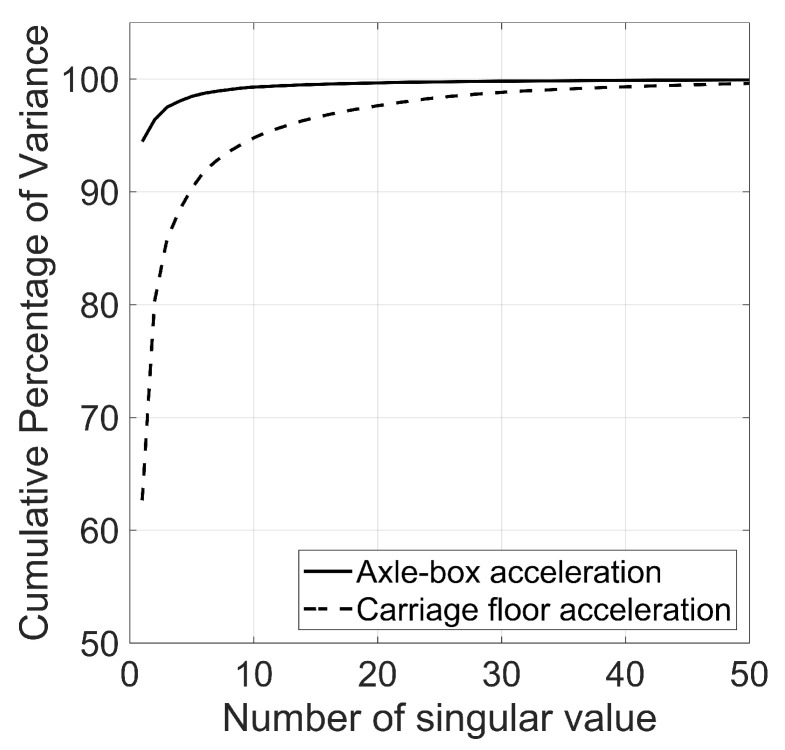
Cumulative percentage of variance for ABA and CFA.

In implementing the algorithm, as previously discussed, the E-RCI will be evaluated using axle-box and carriage floor acceleration data when trains pass through the target section. Figure 8 illustrates the spectrogram cumulative percentage of variance. For this study, the ten highest singular values are chosen for axle-box acceleration (ABA), and the thirty highest singular values are chosen for carriage floor acceleration (CFA). Selecting these specific singular value indices ensures that 95% of the cumulative percentage of variance for both ABA and CFA is captured. 

**Figure 9:**
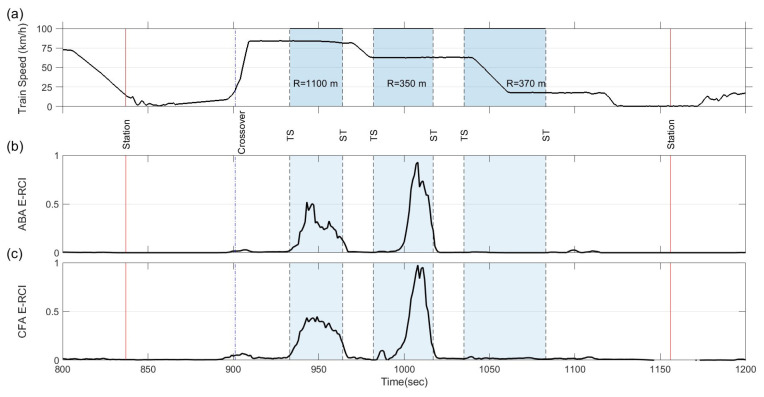
Normalized E-RCI: (**a**) corresponding train speed; (**b**) E-RCI by ABA; (**c**) E-RCI by CFA.

The E-RCI calculated from the acceleration data measured at the axle-box and carriage floor is shown in Figure 9b,c, respectively. These figures show that the E-RCI remains unchanged during the straight sections when the train exits the station and undergoes acceleration. Even when the train passes through the scissor crossover, the wheel–rail impact force does not significantly affect the E-RCI. However, upon entering the first curve with a radius of 1100 m, the E-RCI increases significantly at both measurement points; both of the maximum E-RCI occur at 943 s. As the train exits the first curve, the index returns to a calmer state. Subsequently, upon entering the next curve with a radius of 350 m, the index does not increase immediately. Instead, the index shows a rapid increase until the train reaches the curve′s middle point at 1008 s. 

The E-RCI calculated at both measurement points aligns with the site visit results, accurately representing the range and severity of the rail corrugation. Moreover, the strong correlation between both measurement points on the train indicates the E-RCI′s consistency and reliability in detecting rail corrugations by CFA. In the subsequent paragraph, a case study will be conducted to verify the stability, replicability, and sensitivity of the E-RCI developed. This additional validation will further establish the credibility and applicability of the proposed E-RCI.

## 5. Case Study

In the previous section, the validity of the rail corrugation index calculated based on the CFA was verified. To ensure the stability, replicability, and sensitivity of this index, measurements were conducted on three different train sets, as illustrated in Table 2, serving distinct objectives. Firstly, the CFA data from five different runs of the train set A were used to assess the stability of the E-RCI. These five runs were conducted on the same day under stable weather conditions. This verification aimed to confirm the consistency and reliability of the index within a short time frame. Secondly, train set B was employed to assess the replicability of the E-RCI. Measurements for train set B were conducted one month after those for train set A, assuming that the rail conditions remained unchanged during this period. This analysis sought to validate whether the index could consistently reproduce similar results when applied to different train set measurements. Lastly, the sensitivity of the index was evaluated using train set C. After removing rail corrugations through rail grinding in another section, measurements were taken to gauge the index′s ability to detect changes in rail conditions. 

### 5.1. Index Stability

**Figure 10:**
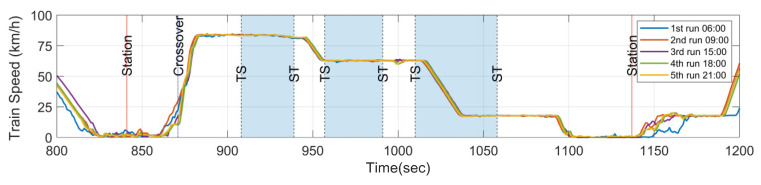
Train speed on five different runs within the target section.

Measuring vibrations on in-service trains can introduce a degree of uncertainty, as it is influenced by factors such as train speed, passenger load, passenger movement, and wind load, among others. However, in this study, the CFA is utilized to evaluate the E-RCI induced by sound pressure and acceleration energy. Fortunately, the metro system under investigation is based on communication-based train control (CBTC), and the train speeds are relatively consistent at identical locations during each run, as shown in Figure 10. As a result, the sound pressure or vibration energy caused by rail corrugation is also expected to be similar due to the consistent train speeds. Therefore, the train speed will not be the significant factor affecting the CFA measurements in this study. 

**Figure 11:**
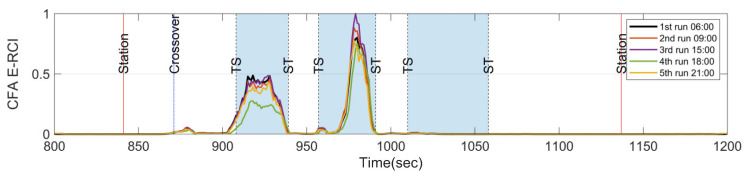
Index on five different runs and repeatability verification on train set A.

In this section, it is essential to note that the measurement instruments, weather conditions, train set configuration, and target section remain consistent (except for wheel wear and severity of rail corrugation over one day). As illustrated by Figure 11, the E-RCIs were derived from five distinct runs using train set A. These runs were conducted during both peak and off-peak times to account for variations caused by passenger presence. The results demonstrate that even with the fixed train set A, the E-RCI calculated by CFA exhibits a highly consistent trend across different runs. While the index may vary slightly during each pass through the target section, upon excluding the maximum and minimum values, the standard deviation of E-RCI peaks remains within the range of 0.023 for the first curve and 0.057 for the second curve. The repeatability of the E-RCI shows a strong correlation in each run, and this confirms the stability and replicability of the developed index for detecting and quantifying rail corrugations.

### 5.2. Index Replicability

In the last section, the stability of the E-RCI index is further verified under controlled conditions. To ensure that specific train set conditions do not influence the acceleration of the carriage floor, the data supervisory and acquisition system was transplanted to train set B. This analysis aims to demonstrate that the E-RCI consistently captures rail corrugation characteristics regardless of the specific train set used.

Here, the target section remains unchanged, as depicted in Figure 5a, and the accelerometer is mounted at the same location on the carriage floor for both train sets. The time difference between the two measurements is approximately one month, as indicated in Table 2. Additionally, it is noteworthy that the replacement of wheels for train set A occurred four months earlier than that of train set B.

**Figure 12:**
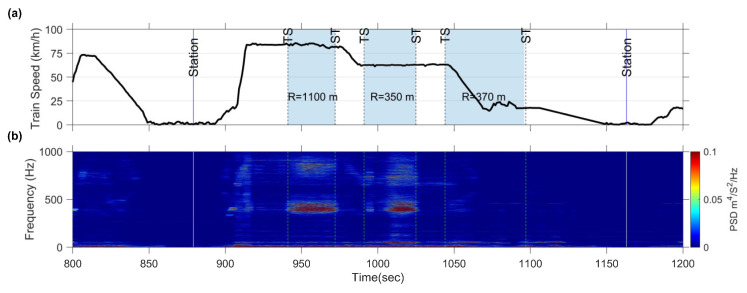
Measured acceleration data PSD spectrogram as train set B passes through the target section: (**a**) train speed of train set B; (**b**) CFA spectrogram of train set B.

**Figure 13:**
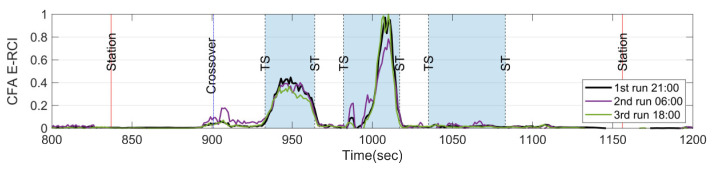
Index reproducibility verification on train set B.

Figure 12 displays the spectrogram of the train set B as it passes through the target section. Upon comparison with Figure 5e, it is evident that the spectrogram of both train sets exhibits the characteristic feature frequencies at approximately 430 Hz and 398 Hz during their passage through the first two curves at the same train speed. Figure 13 presents the E-RCIs calculated for train set B. Notably, the index experiences a significant increase at both the first and second curves, with the trends of both train sets A and B demonstrating high similarity. The standard deviation of E-RCI peaks remains within the range of 0.056 for the first curve and 0.156 for the second curve of train set B. This consistency further validates the effectiveness of the E-RCI in capturing the rail corrugation characteristics, even when different train sets are utilized. 

It is discerned that as the train traverses the same rail corrugation section, a remarkable resemblance emerges in the combination of rail curve radius, wheel vibrations, and the existing wavelength of the rail corrugation. Despite variations in wheel conditions, the resulting exciting frequencies remain consistent. Nonetheless, on a microscopic level, a slight variation of E-RCI between train sets A and B is observed, which could be attributed to differences in the conicity of the wheel tread. This disparity influences the exciting frequency and sound pressure levels, warranting further investigation in the future. However, the most crucial aspect is that measuring different train sets substantiates the viability of E-RCI across various train sets and the ability to eliminate characteristic influencing factors unique to a single train. Nevertheless, using CFA from different train sets as the source material for determining the long-term trend of rail corrugation conditions is not recommended.

Overall, the results obtained from train set B reaffirm the stability and reliability of the E-RCI in detecting rail corrugations, underscoring its practical applicability across different train sets within the same target section.

### 5.3. Index Sensitivity

**Figure 14:**
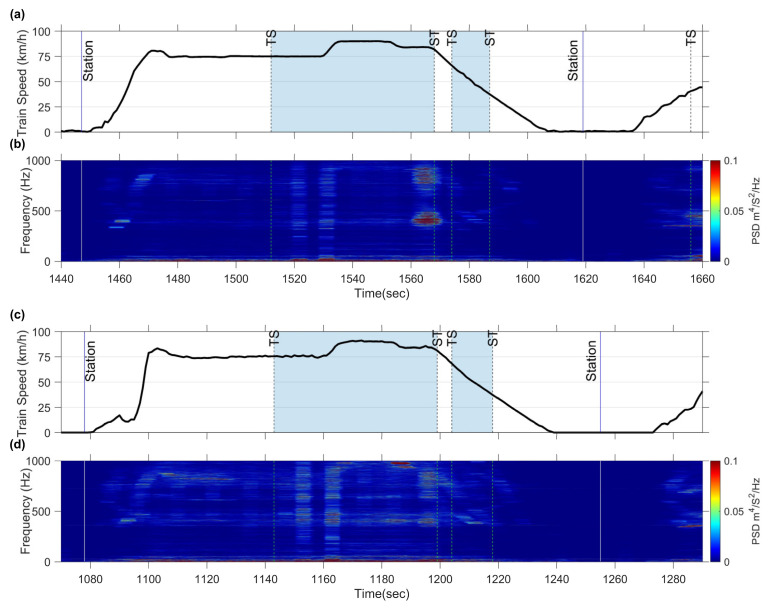
Measured acceleration data PSD spectrogram as train set passes through the rail grinding section: (**a**) train speed of train set B; (**b**) spectrogram of train set B; (**c**) train speed of train set C (after rail grinding); (**d**) spectrogram of train set C (after rail grinding).

The preceding discussion demonstrates the feasibility of calculating E-RCI using CFA and evaluates its stability and replicability across different train sets. Now, the focus will shift to discussing the E-RCI sensitivity in another section, before and after rail grinding. The background data and train speed of this section are illustrated in Figure 14a, encompassing two curves with radii of 800 m each. Rail corrugation was present when train set B was measured; however, it had since been removed through rail grinding when train set C was measured. 

**Figure 15:**
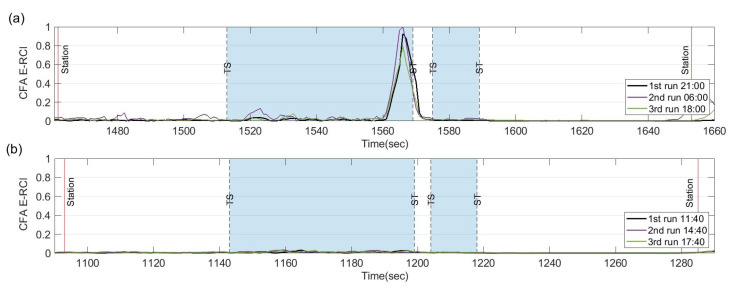
Index sensitivity verification: (**a**) E-RCI calculated by CFA on train set B before rail grinding; (**b**) E-RCI calculated by CFA on train set C after rail grinding.

The CFA measurements were conducted both before and after the rail grinding process on train sets B and C. Figure 14b displays the spectrogram before rail grinding, revealing peak frequencies around 400 Hz and 800 Hz near the ST point of the first curve. However, the peaks rapidly decreased after rail grinding, as illustrated in Figure 14c. Moreover, the E-RCI results, shown in Figure 15, were obtained with $f_{U}$, and $f_{L}$ set at 500 Hz and 300 Hz, respectively. The E-RCI demonstrates significant differences within the grinding section, underscoring the sensitivity of the index developed in this study in detecting variations in rail corrugation.

## 6. Conclusions

The primary goal of this study is to make contributions to enhancing railway operations and infrastructure by introducing a more efficient and effective method for identifying and managing rail corrugation issues. Traditionally, ABA has been widely employed to assess rail corrugation on in-service trains. However, the drawback lies in the significantly higher threshold required for measuring axle-box vibration than vibrations on the carriage floor. This study presents the development of a rail corrugation index that utilizes CFA resulting from sound pressure underneath the carriage. This study develops an effective index to monitor rail corrugations by focusing on the sound-field excitation phenomenon. The conclusions can be summarized as follows:

1. The feature frequency analysis of the PSD spectrogram, obtained through simultaneous measurements of ABA and CFA, demonstrates remarkable consistency. Both acceleration spectrograms share prominent peak frequencies at 430 Hz and 398 Hz, respectively. Moreover, the E-RCI, which accumulates energy within the rail corrugation eigenfrequency range, exhibits a high consistency level. In the first curve, the relatively more significant E-RCI value occurs at the same time point (i.e., 943 s), while in the second curve, they both occur at 1008 s. This provides compelling evidence that the accelerometer mounted on the carriage floor can accurately measure the “sound-field excitation” originating from the wheel–rail contact point.
2. The stability of the E-RCI was assessed through measurements conducted on the same train set within a single day of five different runs. The E-RCI obtained using the CFA consistently exhibited a highly similar trend across five different runs. Upon excluding the maximum and minimum values, the E-RCI peaks’ standard deviation remained within the range of 0.023 for the first curve and 0.057 for the second curve. This shows a remarkable reliability and replicability of the developed index for effectively detecting and quantifying rail corrugations.
3. The replicability of the E-RCI was thoroughly examined by conducting measurements on different trains using the same data supervisory and acquisition system with accelerometers mounted at identical positions. The spectrogram of train set B reveals characteristic frequencies around 430 Hz and 398 Hz within the target section. The E-RCI values also demonstrate a high degree of similarity between the two train sets. The standard deviation of E-RCI peaks remained within the range of 0.056 for the first curve and 0.156 for the second curve of train set B. The comparison demonstrates its ability to capture and characterize rail corrugations, irrespective of the specific train utilized.
4. The sensitivity of the E-RCI was thoroughly examined through CFA measurements conducted both before and after the rail grinding process on train sets. The results clearly exhibit significant differences in the E-RCI within the grinding section before and after the grinding operation. This underscores the sensitivity of the index developed in this study, enabling it to detect variations in rail corrugation effectively.
5. The E-RCI showed slight variations between train sets when passing through the same sections. Therefore, utilizing CFA data from different train sets as the basis for determining the long-term trend of rail corrugation conditions is not recommended until the influence parameters are carefully clarified and thoroughly understood. This will ensure the accurate interpretation and utilization of E-RCI data in rail corrugation management and maintenance strategies.
6. This study has introduced an innovative methodology for rail corrugation detection using CFA, with the principal objective of lowering the detection threshold. The utilization of sound-field excitation on the carriage floor provides valuable information for detecting rail corrugation characteristic frequencies. The study presents an effective measurement technique for identifying rail corrugation areas, which holds the potential to reduce maintenance expenditures and enhance operational efficiency within the railway sector. In the future, conducting long-term continuous measurements to ascertain corrugation amplitudes and subsequently comparing this data with information from corrugation analysis trolleys would be a worthwhile avenue for further investigation.

## Figures and Tables

**Table 1:** Sensors adopted in this study.

Sensors	Specifications	Installed Location
Accelerometer	100 mV/g	Axle-Box
Accelerometer	1000 mV/g	Carriage Floor
GPS antenna	1 Hz	Carriage window
DAQ	NI 9171/NI 9234	Carriage

**Table 2:** Measurement cases.

Train Sets	Runs	Measurement Time	Objective
Set A	1st/6:00, 2nd/9:00, 3rd/12:00, 4th/18:00, 5th/21:00	July, 2022	Stability verification
Set B	1st/06:00, 2nd/12:00, 3rd/18:00	August, 2022	Replicability verification
Set C	1st/11:00, 2nd/14:00, 3rd/17:00	March, 2023	Sensitivity verification

## Data Availability

Not applicable.
